# Genetic diversity, viability and conservation value of the global captive population of the Moroccan Royal lions

**DOI:** 10.1371/journal.pone.0258714

**Published:** 2021-12-28

**Authors:** Kristina Lehocká, Simon A. Black, Adrian Harland, Ondrej Kadlečík, Radovan Kasarda, Nina Moravčíková

**Affiliations:** 1 Institute of Nutrition and Genomics, Slovak University of Agriculture, Nitra, Slovakia; 2 Durrell Institue of Conservation & Ecology, University of Kent, Kent, United Kingdom; 3 Aspinall Foundation, Bekesbourne, Canterbury, United Kingdom; University of Illinois at Urbana-Champaign, UNITED STATES

## Abstract

This study evaluates the diversity of the so-called ‘Moroccan Royal lions’ using genealogical information. Lions are no longer extant in North Africa, but the previous wild population was an important element of the now-recognised northern subspecies (*Panthera leo leo*) that ranged across West Africa, North Africa and the Middle East into India. The remaining captive population of ‘Moroccan Royal lions’ seems to be significantly endangered by the loss of diversity due to the effective population size decrease. The pedigree file of this captive lion population consisted of 454 individuals, while the reference population included 98 animals (47 males and 51 females). The completeness of the pedigree data significantly decreased with an increasing number of generations. The highest percentage of pedigree completeness (over 70%) was achieved in the first generation of the reference population. Pedigree-based parameters derived from the common ancestor and gene origin were used to estimate the state of diversity. In the reference population, the average inbreeding coefficient was 2.14%, while the individual increase in inbreeding over generations was 2.31%. Overall, the reference population showed lower average inbreeding and average relatedness compared with the pedigree file. The number of founders (47), the effective number of founders (24) and the effective number of ancestors (22) were estimated in the reference population. The effective population size of 14.02 individuals confirms the critically endangered status of the population and rapid loss of diversity in the future. Thus, continuous monitoring of the genetic diversity of the ‘Moroccan Royal lion’ group is required, especially for long-term conservation management purposes, as it would be an important captive group should further DNA studies establish an affinity to *P*. *leo leo*.

## Introduction

The lion *Panthera leo* [1] is one of the most charismatic mammalian species, a member of the family *Felidae* yet with an appearance and behaviour that differ considerably from other felid species. Lions have pronounced sexual dimorphism, the males being distinguished by a massive body structure and a mane that extends from head to chest, sometimes covering part of the chest [2, 3]. *P*. *leo* was originally ranged across Africa (except the central Saharan regions), the Middle East, south-eastern Europe (Greece and Bulgaria), central Asia (specifically the Caucasus) and in north-western regions of South Asia (Pakistan and India) [4, 5]. The so-called ‘Barbary lion’ from North Africa was distinctive in being characterised by a massive mane that can be coloured from light to dark and can extend from the head, neck and down to the belly and under the belly to the elbows [6], as well as other morphological features [7].

### Historical population distribution of lions

In past natural history accounts, lion populations had been characterised by geographical groups and thus had been given colloquial names. The Cape lion was found in South Africa, the Barbary lion occurred in North Africa (Morocco, Algeria, Tunisia and Libya) and the Asiatic lion ranged through the Middle East and Arabia through to India [5, 8, 9]. Very early historical records mention the last remnants of European lions in ancient Greece, and a remnant population is thought to have been present in human prehistory in areas adjacent to the western coast of the Black Sea in today’s south-eastern Bulgaria [10]. South of the Sahara, African populations were abundant through the 18^th^ and 19^th^ centuries across West Africa, Central Africa and East Africa. In the 19^th^ and 20^th^ centuries, these various geographical populations across Africa and Asia were allocated subspecies status, based largely on relatively subjective assessments of morphology [10]. In recent years, using molecular studies of lion populations worldwide, a consensus within the scientific community has redefined lions into two distinct subspecies, a northern subspecies *Panthera leo leo* and a Southern subspecies *Panthera leo melanochaita* [11–13].

Perspectives on the conservation of lions have changed significantly over the past two decades. Wild lion populations have rapidly declined in Africa over the past 50 years [14, 15]. The current number of mature individuals of *P*. *leo* is estimated between 23,000 and 39,000. While the current population has a decreasing trend [16], the most critical issues relate to the potential loss of the last isolated micro-populations surviving in West Africa and the similarly endangered small populations in Central Africa. Apart from those, the population of lions in India remains a concern to the International Union for Conservation of Nature (IUCN) [17]. Furthermore, the genetic diversity across lions in the Indian population is very low [13].

The Barbary lion ranged within North Africa’s geographically distinct Maghreb region bounded by the Mediterranean Sea and the Atlantic Ocean to the north and the Atlas Mountains to the south [8], making the population isolated from the rest of Africa by the Sahara [18, 19]. The lions of North Africa have been considered different from other populations [20] due to their distribution, ecology and behaviour [15], as well as specific aspects of their morphology [21]. In modern conservation terms, they would be considered an evolutionarily significant unit [22].

Guggisberg [8] specified that Barbary lions were widespread from the Atlas Mountains to the Mediterranean coast until the 18^th^ century. Persecution resulted in population reduction to remnants in western Morocco, northern Algeria and north-eastern Tunisia by the second half of the 19^th^ century. Several studies indicate the Barbary lions’ populations had been completely extirpated during the early 20th century [23, 24], with some studies suggesting extermination between approximately 1920 and 1930 [8, 21, 25]. Hemmer [21] and Guggisberg [8] reported that in Algeria, Barbary lions survived until the 1890s, as suggested by hunting accounts, reports of catching wild cubs and published photographs of tame lions. The IUCN and others have recognised that this subspecies persisted in Morocco until the 1940s, 1950s and even into the 1960s [8, 10, 15, 21, 25–27].

Barnett et al. [28] reported that some Barbary lions were held captive in menageries in Europe in medieval times. Barbary lions were also a popular showpiece in public zoological gardens in the 1800s [8, 21]. Zoological gardens and circuses in Europe and North America also frequently promoted their lion collections as ‘Barbary’ in the early 20^th^ century [29]. Interest in lions kept in the zoo at Rabat, Morocco, was initiated by German biologists in the 1970s [21] based on a study identifying animals with the physical characteristics of the Barbary lion. The collection was only recognised at that time as essential when the lions were moved from the Royal Palace to a new zoo in Temara, near Rabat. In the 1990s, the IUCN Wild Cat Status Survey and Action Plan indicated that some lions in captivity could be ‘Moroccan Royal lions’, while the wild population was extinct [18, 20]. The best provenance for likely ‘Moroccan Royal lions’ are animals derived from the Moroccan royal collection, originally established from animals captured in the wild by local tribes as tributes to the Sultan, perhaps up until the early 20^th^ century [7].

There are still questions concerning the authenticity of the ‘Moroccan Royal lions’ as the ‘Moroccan Rabat Zoo lineage’ [15, 17]. To date, there has been no evidence to support that the Moroccan King’s lions have maintained the bloodline of the extinct North African Barbary lion. Hemmer [21] stated one of the problems is that some lions in the Moroccan collection had hybridised with other lions from sub-Saharan Africa, potentially added to the collection 70 years ago. Genetic matches among the ‘Moroccan Royal lions’ and the wild Barbary lions have not yet been definitively established [29]. Barnett et al. [28] used museum samples of Barbary lions in their study, but this research was partially limited by the lack of reference samples with a wild origin. However, no living animals have been definitively proven to be of Barbary lion origin in the last three decades.

Captive breeding has enjoyed a recent renaissance in the wider conservation community [30, 31]. Despite the efforts of genetic studies, the evolutionary importance of lions from the Moroccan collection has not yet been fully established, but prudence suggests that it is still necessary to establish an official scientific breeding programme [15]. Yamaguchi and Haddane [7] reported that there are currently only a few lion specimens that can be considered predominantly pure putative representatives of Barbary lions. Black [32] pointed out that there are several lions in European zoos that can be considered partial ‘Moroccan Royal lions’ but cannot necessarily be described as pure-blooded Barbary lions. A studbook labelled as ‘Moroccan Royal lions’ has since been developed for the animals directly descended from the King of Morrocco’s original collection [15, 29] so that, if later proven to be distinct, the animals in this group are conserved.

Speculation about possible current representatives of North African lions in captivity has lasted for decades without the possibility of resolution. It is important to note that to this day it is not known whether the current ‘Moroccan Rabat Zoo lineage’ actually represents wild North African lions, whether they are only partially derived from this wild subspecies or whether they are not really related to wild North African lions [5].

Among the first attempts to solve the problem, the study of Hemmer and Leyhausen [33] was based on preventing the introgression of a foreign allele by determining the external characteristics and checking the features of the skull [20]. This cannot be considered the best solution because the morphological identification of animals living in captivity is influenced by several factors such as nutrition, testosterone levels, climatic conditions, general health and welfare [5, 34].

In recent decades, the relationship between lion populations has been clarified to some extent with the help of molecular technologies. Barnett et al. [28, 35] made initial progress by using mitochondrial DNA (mtDNA) analysis. The results of the mitochondrial analysis of a small sample of analysed King of the Morocco’s lions indicated four haplotypes characteristic of lions from the Central African Republic and Ethiopia, and one lion identical to a haplotype characteristic of lions from north-eastern Sudan [35].

However, even with the use of molecular technologies, there have been several practical limitations, such as the lack of reference samples [36]. In previous studies, including genetic analysis, researchers have been more inclined to the idea that lions from the Rabat Zoo have had a complicated history and are thought to have originated in West or Central Africa [35, 36]. It is important to note that mitochondrial DNA studies were performed on a small number of animals from the collection of the ‘Moroccan Royal lions’. Black [5] stated that the studbook contains 12 founding bloodlines in the entire population [29], but previous DNA studies have analysed only 2 or 3 of these bloodlines [23, 35, 37].

Yamaguchi [38] stated that at present it is not possible to identify accurately living putative representatives of North African lions without comparing individuals to lions that are known to be of North African origin or comparing individuals to samples from all populations of living lions.

#### Pressure on global lion populations

The IUCN [14] reported that wild populations of lions, like those of other large mammalian carnivores, have suffered a serious decline in Africa over the past 50 years. Historically, from observations in North Africa, micro-populations of lions can persist but then suddenly collapse, a situation mirrored today in West Africa in particular, [15], so the conservation concern is real and present. Although the exact causes of lion population decline are not well understood, Riggio et al. [39] reported habitat loss and conflicts with humans as major factors. As populations of any species reduce in size, there is an associated reduction in genetic diversity [40]. Frankham et al. [41] define genetic diversity as diversity at the level of genotypes and alleles present in populations that may be expressed in the form of morphological, physiological and behavioural differences among individuals of that species.

#### Understanding diversity in small populations

Evaluation and description of genetic diversity can be achieved in several ways based on genealogical information. These data place importance on information concerning parental lineage, along with clarification and completeness of pedigrees of bloodlines [42, 43]. Toro et al. [44] asserted that genetic variation is essential to secure advantageous adaptation to changing environmental conditions for populations and pointed out the importance of assessing the diversity in the population to inform the development of conservation strategies. The development of breeding strategies should be based on minimising inbreeding and preventing a further increase in relatedness among animals. Failure to meet these baseline goals results in inbreeding depression that arises from uncontrolled crossbreeding and may result in reduced breeding performance and deterioration in the fitness of individuals and the population as a whole [45]. Pavlík et al. [46] referred to the control of inbreeding as a significant tool for the retention of genetic diversity. Several studies indicate the use of pedigree analysis as a very effective method to evaluate factors such as the inbreeding level [45–48], the loss of genetic diversity [45, 49] and genetic management of endangered populations [50]. Many international programmes, organisations and strategies are addressing the problem of managing species recovery through the use of pedigree, inbreeding and genetic diversity with a focus on mitigating issues of genetic diversity through active genetic management of populations [40].

### Aims of the study

The objective of this study was to evaluate the genetic diversity of the ‘Moroccan Rabat Zoo lineage’ animals currently held captive in various international zoos based on genealogical information. With the current state of lions from the northern group under significant threat in the wild and with a negligible captive population [5], the importance of such animals as captive representatives of the subspecies has become more significant. Black et al. [5, 15, 29] investigated the genetic diversity and viability of the ‘Moroccan Royal lion’ population in several studies focused on conservation methods that can potentially help to prevent significant loss of diversity. Similarly to Black et al. [15, 51], this study examined the vulnerability of ‘Moroccan Royal lion’ populations as well as the need of continued conservation to potentially support this and other remnant populations that may represent North African, West African and Asiatic lions as the collective ‘northern’ subspecies *P*. *leo leo* [12, 13].

## Material and methods

The dataset consisted of 454 individual records of the captive population of ‘Moroccan Rabat Zoo lineage’, which were recorded in Studbooks between 2011 and 2017, namely the studbooks maintained by the European Association of Zoos and Aquaria for the ‘Moroccan Royal lions’.

Our database includes individuals from Black et al. [29] and new data of animals collected from 1969 to 2019. The main source of pedigree data was records from Harland (2017, *personal communication*), who is the author and coordinator of the breeding book containing records from the Rabat Zoo and ‘Western’ zoos (predominantly Europe).

In addition, the database was supplemented with the latest records from Black (2017, *personal communication*) and official publications from zoos [52–56]. For a better understanding of the historical beginnings of lion breeding in Slovakia and the Czech Republic, national zoos in those countries were contacted. Public data from the zoo websites, newsletters, and personal communications with zoo staff were considered (Holečková, Veselá, *personal communication*).

### Pedigree data

The pedigree file consisted of 454 animals (Table 1), including 236 (51.98%) female lions and 218 (48.02%) male lions. Ancestors with unknown parents were considered founders. The reference population consisted of 98 individuals (Table 2) (i.e. living animals currently in zoo collections), divided by sex into a group of females consisting of 51 (11.23%) living individuals and a group of males consisting of 47 (10.35%) living individuals. The reference population represents 19.6% of all ‘Moroccan Royal lions’ kept in captivity and only fertile, living individuals. The database of individuals in pedigree file and reference population consisted of the following variables: individual number, father (sire) number and mother (dam) number for that individual, date of birth of the individual, sex of the individual and individual order in generations. Individuals who have known pedigrees form a kind of the imaginary nucleus of that population and are therefore the subject of analysis.

**Table 1:** Number of animals according to the studbook 2011.

Location	Number of individuals
Austria	4
Cuba	3
Czech Republic	54
France	36
Germany	89
Israel	2
Italy	1
Morocco	132
Slovakia	3
Spain	31
Switzerland	3
United kingdom	31
Unknown	47
USA	14
Vietnam	4

**Table 2:** Number of living individuals in the world according to the studbook 2017.

Location	Number of individuals
Austria	2
Czech Republic	13
France	12
Germany	13
Italy	1
Morocco	41
Slovakia	4
Switzerland	1
United kingdom	10
Unknown	1

### Analysis of genetic diversity

Individuals from the Moroccan Rabat Zoo collection without complete parental information or date of birth and dead cubs at an early age were not considered in the analysis. However, pedigree and morphological data of these animals may be important for future analyses. The original dataset included five columns: father, mother, place of birth and date of birth (if known), number of progeny. A column identifying animals from the reference population was added to the original dataset. The final dataset was prepared by using the SAS Enterprise Guide 8.1 software according to the requirements of the Endog V4.8 software [57], which was used to calculate pedigree completeness and pedigree-based diversity parameters in subsequent analyses. Each of the analysed pedigree-based diversity parameters was calculated separately for the reference population and the pedigree file.

#### Pedigree completeness

Four parameters were used to evaluate the pedigree completeness. First, ‘the maximum number of generations traced’ represents the number of generations separating the individual from its furthest ancestor. Second, ‘the number of fully traced generations’ was used to determine the number of generations separating the offspring of the furthest generation where both ancestors of the individual are known. Ancestors with an unknown parent are considered founders (generation 0). Third, the ‘equivalent complete generations’ was calculated according to Maignel et al. [58] as the sum over all known ancestors of the terms calculated as the sum of *(1/2)*^*n*^, where *n* is the number of generations dividing the individual to each known ancestor. The last parameter was the ‘pedigree completeness index’ (PCI), which was calculated according to MacCluer et al. [59] for individuals as the harmonic mean of paternal and maternal lines:

$$PCI=\frac{2{C_{p}C}_{m\;}}{C_{p}+C_{m}}$$ (1)

where C_*p*_ and C_*m*_ represent contributions from the paternal and maternal line, but individually:

$$C=\frac{1}{d}\sum_{i=1}^{d}g_{i}$$ (2)

where *g*_*i*_ is the share of known ancestors in generations *I* and *d* is the number of generations counted.

### Pedigree-based diversity parameters derived based on the common ancestor

The genetic diversity was initially estimated by calculating the pedigree-based parameters derived from a common ancestor (effective population size *N*_*e*_, inbreeding coefficient of an animal *F*, individual increase in inbreeding per generation *ΔF*_*i*_, average relatedness *AR*) [45]. Wright [60] defined the inbreeding coefficient of an animal (*F*_*i*_) as the probability that an individual has two identical alleles from one common ancestor. This coefficient assumes that the analysed individuals and their common ancestors do not originate from the mating of relatives; *F* is calculated according to the equation:

$$F_{x}=\sum 0.5^{n_{1}+n_{2}+1}+(1+F_{a})$$ (3)

where *n*_*1*_ is the number of generations from the individual X to the common ancestor on the father’s side, and *n*_*2*_ is the number of generations from individual Y to the common ancestor on the mother’s side. Furthermore, *1 + F*_*a*_ represents the correction factor of the inbreeding coefficient of the common ancestor in the path [61].

Toro et al. [44] defined inbreeding as one of the principal genetic factors that can endanger the life of the population in a short time interval. The availability of information on the pedigree and its depth influence the estimation of the inbreeding coefficient, while the increase in inbreeding per generation (*ΔF*) is influenced by the relative increase between generations. The increase in inbreeding (*ΔF*) was calculated according to Gutiérrez et al. [62] as follows:

$$\Delta F=\frac{F_{t}-F_{t-1}}{1-F_{t-1}}$$ (4)

where *F*_*t*_ and *F*_*t-1*_ are the averages at t and *t-1* generations, respectively.

According to Gutiérrez et al. [62], the average relatedness coefficient (*AR*) expresses the probability that a randomly selected allele from all populations in a pedigree belongs to a given animal. *AR* is an alternative or complement to the inbreeding coefficient. It is used to express long-term inbreeding within a population because it reflects the percentage of complete pedigree originating from founders at the population level [62].

The effective population size (*N*_*e*_) is used to express the individual increase in inbreeding (*ΔF*). Hill and Zhang [63] defined the effective population size as the size of an idealised population where there is an equal increase in drift or inbreeding per observed generation. *N*_*e*_ can be defined as the number of reproductive individuals raised in an idealised population, within which all individuals are of the same sex and selfing is permitted. In this case, the observed population shows an equal decline in pedigree-based genetic diversity [64]. Several studies have mentioned that the estimation of effective population size could be used to quantify the loss of genetic variation and to estimate the degree of inbreeding [64–67]. Lewis et al. [68] estimated the measure of genetic diversity loss and determined the threat posed by inbreeding based on *N*_*e*_ values. When the effective population size is > 100, there is a very slow decline leading to maintenance of most genetic diversity in a given population. However, if *N*_*e*_ is < 50, such a population is at high risk because of the harmful effects of inbreeding. Gutiérrez et al. [69] reported that *N*_*e*_ has a straightforward relationship to the level of inbreeding, genetic fitness and the level of loss of genetic variability owing to random genetic drift.

The effective population size of the Moroccan lion (‘Moroccan Royal lions’) group was estimated from pedigree data from the individual increase in inbreeding proposed by Gutiérrez et al. [70] and from the paired increase in co-ancestry [71]. *N*_*e*_ as proposed by Gutiérrez et al. [70] can be estimated from an individual increase in inbreeding (*ΔF*) by averaging *ΔF* of *n* individuals included in a given reference population as:

$$\bar{N_{e}}=\frac{1}{2\bar{\Delta F}}$$ (5)

Cervantes et al. [71] mentioned the estimation of *N*_*e*_ from the increase in co-ancestry for all pairs of individuals *j* and *k* (Δ*c*_*jk*_) in a reference population. Thus, *N*_*e*_ was calculated based on co-ancestry as:

$$\bar{N_{ec}}=\frac{1}{2\bar{\Delta c}}$$ (6)

while the parameter $\bar{\Delta c}$ was computed as:

$$\Delta c_{jk}=1-\sqrt[{\frac{\left(g_{j}+g_{k}\right)}{2}}]{1-c_{jk}}$$ (7)

where *c*_*jk*_ is the inbreeding value corresponding to an offspring from *j* and *k*, and *g*_*j*_ and *g*_*k*_ are the discrete equivalent generation of individuals *j* and *k*.

Due to the limited number of animals per zoo, *N*_*e*_ was calculated only for the reference population.

#### Generation interval

The generation interval is defined as the average age of parents at the birth of their offspring. This definition is based on the contribution of parental age classes to newborn offspring. The average age of the parents is calculated as the sum of the age at birth of the offspring weighted by the increment of each age class to the newborn offspring [63, 72]. The generation intervals were calculated using the Endog V4.8 [57] software as the average age of parents at the birth of their offspring reared for reproduction [73] as well as the average age of parents at the birth of their offspring (regardless of further use of individuals) [74]. The generation interval was calculated for four pathways: father-son, father-daughter, mother-son and mother-daughter.

#### Pedigree-based diversity parameter derived from the probability of gene origin and genetic diversity loss

Lacy [74] defined the number of founders (*f*) as ancestral individuals with unknown parents or with an unknown genetic association with other animals in the pedigree in addition to its progeny. If all founders contribute alike, the number of founders would be equal to the effective number of founders [40]. According to Boichard et al. [75], the effective number of founders (*f*_*e*_) was evaluated as the number of founders with equal contribution, which would give the same amount of genetic diversity that is present in the current population. The effective number of founders was calculated as:

$$f_{e}={\left[\sum_{k=1}^{f}q_{k}^{2}\right]}^{-1}$$ (8)

where *q*_*k*_ represent the probability of gene origin of *k* ancestors.

The effective number of ancestors (*f*_*a*_) was defined as the minimum number of ancestors needed to explain the genetic diversity in the reference population [74]. The effective number of ancestors derived from pedigree was estimated as:

$$f_{a}={\left[\sum_{j=1}^{a}q_{j}^{k}\right]}^{-1}$$ (9)

where *q*_*k*_ represents the marginal contribution of an ancestor *j*—the genetic share made by an ancestor—that is not explained by other ancestors selected previously. Boichard et al. [75] reported that the effective number of ancestors represents the recent bottleneck and thus is partly responsible for the loss of allelic diversity of the offspring.

Founder genome equivalents (*f*_*g*_) according to Lacy [74] present the number of founders that would be expected to provide the same level of genetic diversity in the evaluated population if the founders were represented equally and there was no loss of alleles. This parameter based on pedigree was estimated by the Caballero and Toro [76] algorithm:

$$f_{g}={\left[\sum_{j=1}^{N_{f}}\left(\frac{p_{j}^{2}}{r_{j}}\right)\right]}^{-1}$$ (10)

where *N*_*f*_ is the number of founders, *p*_*j*_ represents the contribution of the founder *j* and *r*_*j*_ is the retention of alleles. Lacy et al. [77] reported that the founder genome equivalents as reflecting the unequal contributions of founders, bottleneck and random loss of alleles due to genetic drift.

The decline in population size in wild or domestic species is often accompanied by a significant loss of genetic diversity. Kadlečík et al. [40] pointed out that it is important to ensure the adaptive potential of the species and to prevent the occurrence of inbreeding depression over a long time. The loss of genetic diversity (GD) was inferred from parameters *f*_*a*_ and *f*_*g*_. Furthermore, the estimation of the total genetic diversity of the reference population used the formula from Lacy et al. [77]:

$$GD=1-\frac{1}{2f_{g}}$$ (11)

The genetic diversity loss owing to bottleneck and genetic drift in the population was estimated as 1—GD. The amount of genetic diversity in the reference population considered to be a loss of diversity due to unequal founder contributions (GD*) was estimated according to Lacy et al. [77] as:

$${GD}^{*}=1-\frac{1}{{2f}_{e}}$$ (12)

As stated above, 1—GD* indicates the loss of genetic diversity due to unequal founder contributions. The difference GD*—GD measures the loss of diversity by genetic drift amassed over non-founder generations, as an estimate by Caballero and Toro [76].

#### Genetic structure of the population based on pedigree data

The relatedness among individuals in the reference file was expressed through a relationship matrix calculated according to Wright [78] and visualised in the form of a heatmap. Subsequently, the genetic relationships of individuals within the evaluated population were also tested by principal component analysis (PCA) using the R package qplots2.

Wright’s fixation index (*F*_*ST*_) was used to describe the level of genetic differentiation between subpopulations defined according to individual countries where animals are currently bred. Thus, subpopulations correspond to the national-level organisations involved in the conservation of ‘Moroccan Royal lions’ worldwide. The *F*_*ST*_ index can theoretically range from 0 to 1 [79–81], where a value close to 0 indicates a very high level of genetic similarity between subpopulations as a result of a high proportion of common ancestors occurring in the pedigrees.

## Results and discussion

### Pedigree completeness

According to Lacy [74], genealogy analysis is generated from the knowledge of the founders of the population. Founders are individuals with unknown or estimated ancestors that gave rise to the current population. Ko and Nielsen [82] defined pedigree as a chronological record containing information about the genealogical relationships among individuals. This record usually contains information across three to five generations. Our dataset consisted of a maximum of seven generations of ancestors, similar to a previous analysis conducted on the American bison (*Bison bison*) reported by Skotarczak et al. [83].

**Fig 1:**
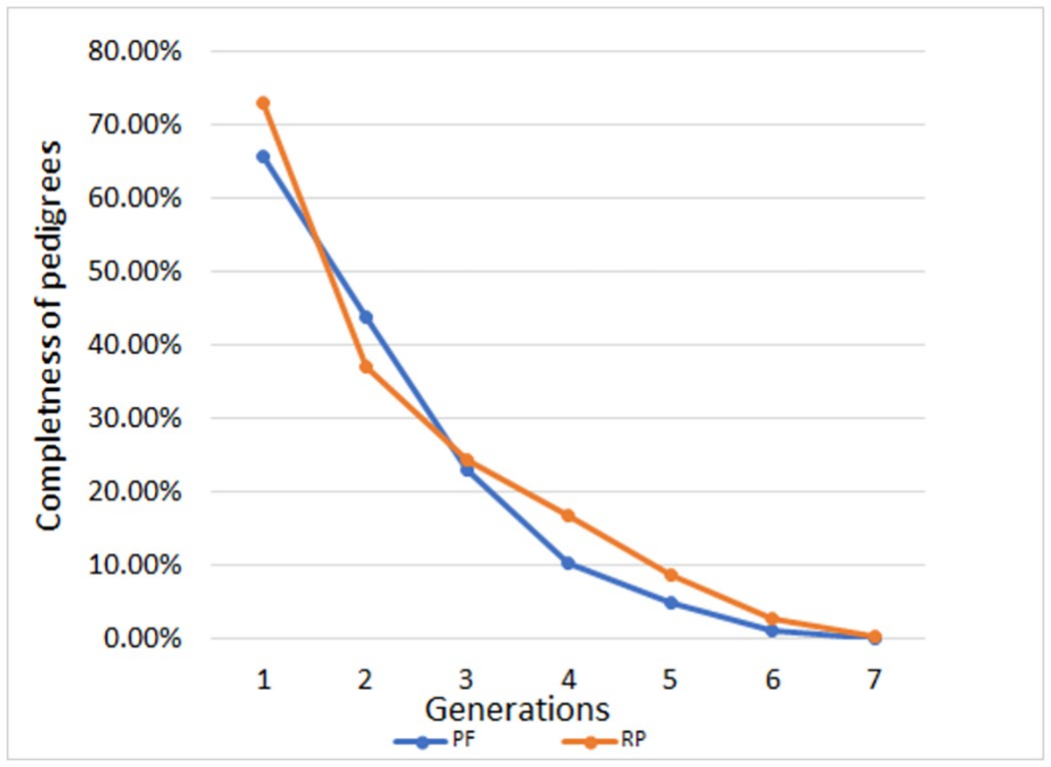
Pedigree completeness index (MacCluer et al. [59]) for the captive ‘Moroccan Royal lions’ population by generations. PF—pedigree file, RP—reference population.

The results of the pedigree completeness index for the captive ‘Moroccan Rabat Zoo lineage’ population are shown in Fig 1. This visualisation indicates that as the number of ancestor generations increases, the percentage of known ancestors decreases. The highest percentage of pedigree completeness was achieved in the first generation of the reference population (> 73%). The average completeness index for the last five generations showed a higher proportion of known ancestors in the reference population (32%) compared with the pedigree file (30%). As expected, we also observed a higher maximum number of known generations in the reference population (2.66) compared with the pedigree file (1.75). Maignel et al. [58] defined this indicator as the number of generations that separate the offspring from the most distant known ancestor. Due to the fact that the quality of pedigrees is given by the number of known ancestor generations, the overall quality of the tested dataset was good. Previous studies have shown that the accuracy of genetic diversity analysis depends on the depth and completeness of the pedigree. The lower maximum number of known generations and pedigree completeness index in the pedigree file compared with the reference population indicate that the level of pedigree completeness has increased in recent years.

The average number of complete generations observed for the ‘Moroccan Royal lions’ population pointed to the decrease in pedigree quality compared with other livestock [46, 84] and wild animal species [83]. The number of complete ancestor generations in the pedigree file was 1.25, while the reference population reached 1.04. The equivalent number of ancestor generations reached higher values (around 1.5) in both groups.

In general, the results showed a significant incompleteness of pedigrees for an observed population. However, increased values of pedigree completeness indices in the reference population point to a gradual improvement in the quality of pedigree information. Although it is well known that the pedigree data and studbook of the ‘Moroccan Rabat Zoo lineage’ is not accurate for all historical stages, it provides valuable information about the current population of the ‘Moroccan Royal lions’. From a genetic point of view, if the molecular data are not available, the studbook is a valuable source of information, mainly regarding a small population of endangered wild animals. In the effort to preserve genetic diversity, each individual is important because it contributes to the population gene pool. To preserve the valuable gene pool of endangered lion populations, including ‘Moroccan Royal lions’, it is possible in the future to use a certain form of crossing, similarly to the restoration process that has been successfully applied in the Przewalski’s horse captive breeding programme [7].

### Pedigree-based diversity parameters derived based on the common ancestor

Intensive selection and reuse of validated sires and dams can result in an increase in the level of inbreeding and a decline in genetic diversity because future generations will carry predominantly alleles derived from a reduced number of parents. In addition, the increase in inbreeding is often accompanied by various undesired effects such as inbreeding depression, population fitness decline and a higher frequency of inherited diseases [85]. Thus, the level of inbreeding is considered one of the most important factors that threaten the survival of wild populations in the short term and the long term [44]. Another pedigree-based parameter that is often used to describe the effect of relative mating on the population gene pool is the average relatedness coefficient (*AR*), which represents the genetic contribution of founders to the population diversity. *AR* is often used as an alternative or complement to the inbreeding coefficient because it expresses long-term inbreeding in the form of the percentage of a complete pedigree originating from founders at the population level [62].

The calculation of individual inbreeding and average relatedness in the tested population point to more intense in inbreeding in the pedigree file (*F* = 5.43%, *AR* = 5.42%) compared with the reference population (*F* = 2.14%, *AR* = 3.43%), which reflects the efforts of breeders to reduce the proportion of related animals in the current mating plans. The highest intensity in inbreeding in the pedigree file also confirmed the value of *ΔF* (3.50%), which showed an increase in inbreeding per generation. Based on these results, a continuous increase in the inbreeding and a gradual decline in genetic diversity in the future can be expected.

To evaluate more precisely the intensity of inbreeding in the ‘Moroccan Royal lions’ population, the individuals were divided into three categories according to the degree of inbreeding. In addition, the level of inbreeding was calculated separately for males and females (Table 3). The highest proportion of individuals were in the first class (*F* ≤ 0). However, this group consisted of individuals or ancestors that are no longer involved in reproduction. This group also included individuals that had incomplete pedigrees, considered genetically pure but with unknown basic ancestors. Overall, 10.2% (reference population) and 7.71% (pedigree file) of animals were included in the second class, representing animals with inbreeding from 1% to 13%. The third class contained the most inbred animals (*F* ≥ 13%-37.5%) in the analysed population. For the reference population, only 6.12% of the animals were involved in this class, while for the pedigree file, up to 14.98% of the animals reached an *F* value of ≥ 13%-37.5%. In the reference population, there was higher individual inbreeding and average relatedness for males compared with females. On the contrary, in the pedigree file, females showed a higher level of individual inbreeding compared with males (Table 3), probably because of the limited number of reproductively active females that were able to produce offspring. Currently, reproduction problems are eliminated mainly as a result of efforts to reduce inbreeding by the utilisation of less related animals in mating programmes. Considering the risk of inbreeding increase in future generations, the optimisation of mating plans for individuals with an *F* value > 1% will be essential.

**Table 3:** Indicators of diversity derived from common ancestors in the pedigree file (PF) and the reference population (RP) for captive ‘Moroccan Royal lions’.

Indicators		RP	PF
Inbreeding coefficient (*F*)		2.14%	5.43%
Coefficient of average relatedness (*AR*)		3.43%	5.71%
*F* by sex		2.24% ♂	4.70% ♂
		2.05% ♀	6.16% ♀
*AR* by sex		3.56% ♂	5.73% ♂
		3.29% ♀	5.68% ♀
Representation of individuals in classes of *F*	*F* ≤ 0	82	351
	*F* = 1–13%	10	35
	*F* ≥ 13–37.5%	6	68
Individual increase in inbreeding (*ΔF*)		2.31%	3.50%

Effective population size is an important parameter for assessing genetic diversity in animal populations. Knowledge of the effective population size provides relevant information needed to monitor genetic diversity, especially in vulnerable and small populations [60]. According to Gutiérrez et al. [69] and Cervantes et al. [71], *N*_*e*_ was estimated based on the individual increase in inbreeding ($\bar{N_{e}}$) and increase in co-ancestry ($\bar{N_{ec}}$). Due to the limited number of animals per zoo, *N*_*e*_ was calculated only for the reference population. $\bar{N_{e}}$ derived from individual inbreeding increase was 22.69 animals, whereas $\bar{N_{ec}}$ reflecting an increase in co-ancestry was only 14.02 animals. According to these values, the population of ‘Moroccan Royal lions’ can be described as critically endangered due to the loss of diversity because the effective population size is less than 50 animals [68]. As shown by Lewis et al. [68], if *N*_*e*_ < 50, such a population is at high risk of extinction because of the harmful effects of inbreeding. Thus, the obtained low *N*_*e*_ estimates also point to the high rate of average relatedness among animals as well as the intensity of inbreeding. Similarly, Frankham et al. [41] stated that a population is resistant to the negative effects of inbreeding if its effective size is > 50 animals, while a size up to 500 individuals is necessary to maintain the long-term diversity and evolutionary potential of a population. For this reason, it is important mainly in the case of a small population of endangered animals, such as ‘Moroccan Royal lions’, to preserve genetic diversity by increasing its effective size, balancing the contribution of individual ancestors and preventing mating of closely related individuals.

#### Generation interval

Gutiérrez et al. [70] defined the generation interval as the average age of parents at the birth of offspring who will be included in reproduction. The generation interval represents the time needed for replacement of one generation with the next. The generation interval is a crucial parameter that provides information on the structure of populations as well as the state of genetic diversity because it is equal to the reciprocal of the total long-term genetic contribution per year. For endangered populations, the generation interval is important mainly due to its relation to the rate of inbreeding. It is influenced by various environmental and species-specific factors and linked to the reproductive abilities of the population. In this study, the generation interval was calculated for the four paths of transmission (father-son, father-daughter, mother-son, mother-daughter) according to Falconer and Mackay [85] separately for the pedigree file and reference population. The results are shown in Tables 4 and 5, respectively.

**Table 4:** The generation interval (years) in the pedigree file.

	n	$\bar{x}$	SD
Father-son	10	8.01	3.33
Father-daughter	18	6.95	3.50
Mother-son	8	6.44	2.83
Mother-daughter	13	7.13	3.96
Total	49	7.13	3.43

$\bar{x}$—average; SD—standard deviation.

**Table 5:** The generation interval (years) in the reference population.

	n	$\bar{x}$	SD
Father-son	4	10.14	3.05
Father-daughter	3	6.67	1.76
Mother-son	4	6.78	3.20
Mother-daughter	2	7.04	5.94
Total	13	7.83	3.30

$\bar{x}$—average; SD—standard deviation.

The father-son path had the longest generation interval in both the pedigree file and the reference population (8.01 and 10.14 years, respectively). These results are consistent with the biological expectations, as lions are territorial and live in groups. In the wild, the male also has several females that produce offspring (4–6 cubs/female/year). Crandall [86] reported that the ageing process in males begins around 10–15 years of age. In the wild, lions live up to 15 years. In captivity, they can live up to 10 more years. However, in captivity, it is not possible to produce such a large number of offspring due to reproductive problems often associated with females, together with the length of gestation (100–120 days). For example, in jaguars (*Panthera onca*), Gonzáles et al. [87] observed a longer generation time for females than for males.

In the reference population, the generation interval—ranging from 6.67 to 10.14 years—was relatively long, probably as a consequence of the long-term use of certified males in reproduction (fertile, proven by progeny). Another reason for such a generation interval is a relatively small population. Hemmer [21] reported much lower values (3.75 years) of the generation interval within 20 generations, which separated the ‘Moroccan Royal lions’ observed in 1974 from wild lions captured in 1899. Hemmer indicated that these low values would provide numerous opportunities for hybridisation options with lions that are not Barbary lions. Black et al. [15] disputed Hemmer’s [21] suggestion and instead indicated comparable values of the average generation interval for ‘Moroccan Royal lions’ ranging from 6.6 to 10 years from the Moroccan Royal Studbook, and those conclusions appear to be strongly corroborated by this study. Halo et al. [84] stated that for the small population focusing on bloodline preservation, long generation intervals are desirable because they minimise an increase in inbreeding. Several studies suggest the similarity between the generation interval of ‘Moroccan Royal lions’ and the birth age (6.5 years) for wild lions [15, 88].

The generation interval of dams was 6.44–7.13 years (Tables 4 and 5). These values are consistent with previous studies in wild female lions (6.5–8 years) [15, 20, 88]. Based on our results, we can assert that the long generation interval indicates a slow rate of genetic change. In our case, the generation intervals are most likely strongly affected by the population size and the health status of the individuals, both of which are related to the level of individual inbreeding. The long-term survival of the ‘Moroccan Royal lions’ depends on sufficient genetic variation for adaptability and fitness [89].

#### Pedigree-based diversity parameter derived from the probability of gene origin and genetic diversity loss

Assessment of genetic diversity using parameters derived from the probability of gene origin describes the diversity of populations after a small number of generations, although over a longer period, inbreeding coefficients and *N*_*e*_ are considered the most important parameters for assessing diversity [74]. The *f*_*e*_ parameter is used to determine whether there is a balanced contribution of founders in a population; thus, it describes the loss of genetic diversity in the population as a result of the unequal contribution of founders [75]. The *f*_*g*_ parameter reflects the proportion of genetic variation stored in the gene pool, especially in small populations. This parameter is usually used to estimate genetic variability loss due to unequal founder contribution or random genetic drift. The *f*_*g*_/*f*_*e*_ ratio estimates the effect of genetic drift and founder contributions to the genetic diversity of population, so lower ratios are connected with a higher incidence of genetic drift [75]. *f*_*a*_ is expressed as the number of equally contributing ancestors who are expected to provide the same amount of genetic diversity as in the evaluated population [75]. This parameter is defined by the marginal contribution of each ancestor, while *f*_*e*_ explains whether a given gene present in the founders is still present in the evaluated population [75, 74]. The *f*_*a*_*/f*_*e*_ ratio indicates the influence of bottleneck in the population and is therefore important in determining the population history [74].

All parameters computed based on the analysis of gene origin probability are summarised in Table 6. Boichard et al. [75] stated that these parameters are suitable indicators to describe a population structure characterised by a small number of known generations. The reference population had more founders (47); there were only 34 founders in the pedigree file. There was a similar trend for the effective number of founders and the effective number of ancestors, where the reference population reached higher values compared with the pedigree file. The rate of *f*_*g*_/*f*_*e*_ describing the effect of genetic drift reached higher value in the pedigree file (1.17) compared with the reference population (0.64). The *f*_*a*_*/f*_*e*_ ratio was similar in the pedigree file and reference population (0.92), a finding that points to the strong effect of genetic drift on the population gene pool because the most important ancestors were relatively old [40]. Eight founders were needed to explain 50% of the genetic diversity in the reference population, whereas in the pedigree file this was only four founders. Thus, the obtained results indicate the excessive use of a small number of verified sires as parents of future generations, which to some extent explains the loss of diversity in the evaluated population. The lower *f*_*a*_*/f*_*e*_ ratio compared with the *f*_*g*_ */f*_*e*_ ratio indicates a loss of genetic variability in the population due to the stronger effect of genetic drift than bottleneck. The total genetic diversity loss was 3.30% in the reference population. The proportion of genetic diversity lost due to the unequal use of founders was 2.10%, and 1.20% of genetic diversity loss was caused by random genetic drift.

**Table 6:** Indicators based on the probability of gene origin in the pedigree file (PF) and the reference population (RP) for captive ‘Moroccan Royal lions’.

Indicators	PF	RP
Number of individuals (*n*)	454	98
Number of founders (*f*)	34	47
Effective number of founders (*f*_*e*_)	13	24
Effective number of ancestors (*f*_*a*_)	12	22
Effective number of genomes (*f*_*g*_)	15.22	15.42
Number of ancestors explaining 50% of genetic diversity	4	8

Our results revealed that the ‘Moroccan Royal lions’ population has suffered a continual loss of genetic diversity mainly due to the unequal founder contribution. In lions as well as in other species, the global diversity decline has been largely due to conflicts between humans and wild animals and, consequently, the loss of habitat [90, 91]. Whitham et al. [92] stated that without genetic diversity, the resilience of animals and the ability to adapt to stochastic events decreases significantly in individual species. Dures et al. [91] reported that there is an urgent need to optimise efforts for the conservation and management of lion populations.

#### Genetic structure of the population based on pedigree data

**Fig 2:**
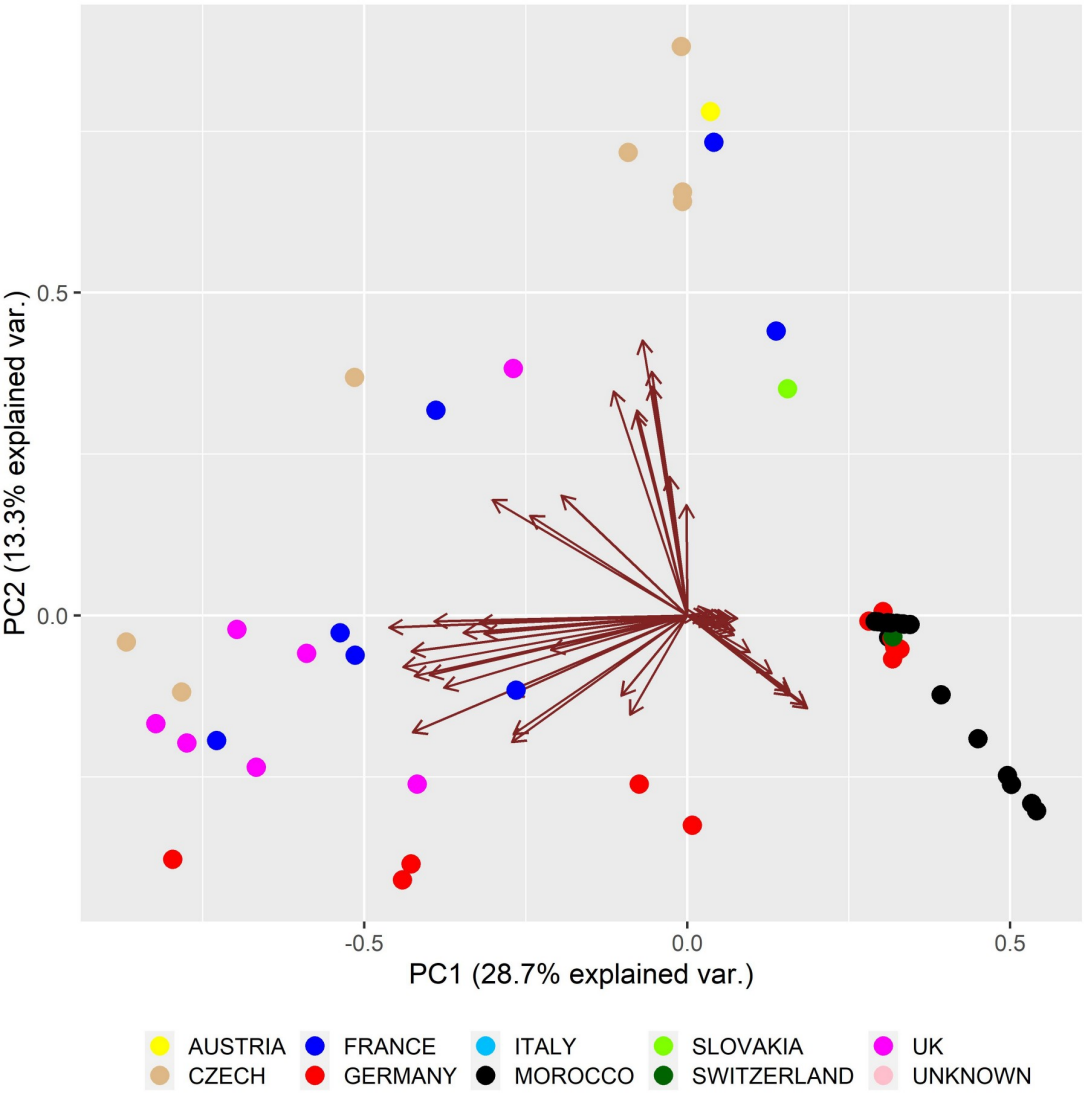
Diagram showing the structure of the pedigree file based on principal component analysis.

Fig 2 shows the substructure of the population derived from relationships among individuals in the reference population based on the PCA. The first two principal components (PCs) explained 42% of the total variability stored in the dataset, thus providing a reliable view to the substructure of reference population. The arrows reflect the effect of each individual on genetic variability conserved in the analysed dataset when the effect of an individual is proportional to the angle between the arrows. If the variables represented by individuals were highly associated, the angle between the arrows was as small as possible. The length of PCs in Fig 2 refers to the amount of variance contributed by the PCs: the longer the PC length, the greater the variance contributed and well represented in space [93]. Individuals are colour coded, with different colours representing the region in which they are currently bred.

From the biplot, the country of origin of individuals is only partly associated with cluster formation and reflected mainly by the intensity of transfer among countries and the breeding programme of the ‘Moroccan Royal lions’. Thus, the distribution of individuals into the groups reflects, in particular, their genetic background. The low number of founders and genetic relatedness among animals in the evaluated population (reference population *f*_*g*_ = 15.42; pedigree file *f*_*g*_ = 15.22) resulted in formation of three clusters.

As can be seen in Fig 2, there has been intense transfer between Morocco, Germany and Switzerland, as evidenced by the studbook. In addition, individuals originating from Morocco are located in separate clusters due to the fact that they formed the breeding core for the ‘Moroccan Royal lions’ in Europe. In Morocco, breeders keep their own studbook and individuals who come from Morocco often do not have known parents or close relatives. This fact has greatly influenced the results of this analysis, which should be interpreted with caution. The relatively intensive exchange of animals was reflected in the creation of a separate cluster consisting of French, German, United Kingdom and Czech Republic zoos. The third cluster consisted of animals from the Czech Republic, Slovakia, Austria and France. In the past, individuals from the Czech Republic had been transported to Austria and Slovakia, as stated in the studbook; therefore, these countries are in a common group.

The Czech zoos, especially the Olomouc Zoo, in the past had been known for their excellent rearing of cubs from proven males with excellent offspring, which was reflected in their intensive transport to other countries. The founders of breeding in the Olomouc Zoo originated from Germany, while in Dvůr Králové Zoo the breeding pair comprised of individuals from Morocco (Veselá, Holečková, *personal communication*).

The issue of ‘Moroccan Royal lions’ has been addressed by several authors. Black et al. [29] dealt with pedigree analysis in their study, but the database included older data that did not correspond to the current state of ‘Moroccan Royal lions’ genetic diversity. Black [5] dealt with the genetics of the ‘Moroccan Rabat Zoo lineage’ and the conservation of putative descendants in captivity. Barnett et al. [35] showed, based on the mitochondrial DNA (mtDNA) analysis, that ‘Moroccan Royal lions’ form a common cluster with lions from Central Africa and the gene pool of those animals has been affected by introgression. However, their study considered only a limited number of individuals that originated from North Africa; therefore, their results should be interpreted with caution. Black [5] presented possible hypotheses explaining such results. One was that the Royal Moroccan Collection did not originate from North Africa. Considering efforts to maintain a separate group for decades, in our view this alternative is untested and unlikely. Another relevant hypothesis that has not been tested was based on the assumption that Moroccan lions have haplotypes corresponding to Central Africa and, therefore, belong to one large population (North Africa, West Africa, Central Africa and India). However, this hypothesis cannot be tested due to small number of museum reference samples that carry all the original haplotypes of the North African populations. Burger and Hemmer [94] presented further analysis based on cytochrome b sequence. DNA was obtained from cubs with parents from the Moroccan collection (Zoo Neuwieda, Germany). Their results indicated that those individuals are more related to the Asian lions (*Panthera leo persica*) than the sub-Saharan lions. However, as presented by Yamaguchi [38], in the absence of a comparison of that sequence with ancient DNA of the Barbary lion, it cannot be confirmed that this cub represents a true wild descendant of the North African lion. At the same time, sequences presented by Burger and Hemmer [94] did not include individuals representing regions such as the Sahel. Therefore, the difference could be caused by comparison with genotypes from other African regions. In addition, Black [5] reported that the mtDNA haplotype of cubs from Neuwieda has not yet been found in any wild lion populations. Based on this, the author assumed that there is a possibility that genetic traits lost from wild populations may have been conserved in the captive animals. Barnett et al. [35] found, based on a small number of mtDNA isolates, that the genetic characteristics of Moroccan lions differ from other lions of known origin. Therefore, it may be appropriate to perform further studies to confirm these claims and, where appropriate, to compare the results with a wider sample of individual lions. Bertola et al. [11] reported that lions in the Central African Republic, Ethiopia and Sudan are important for the conservation of the genetic diversity of the lion in general and the northern subspecies in particular. In our view, this confirms the importance of preservation and restoration of the ‘Moroccan Royal lions’ population in zoos.

**Fig 3:**
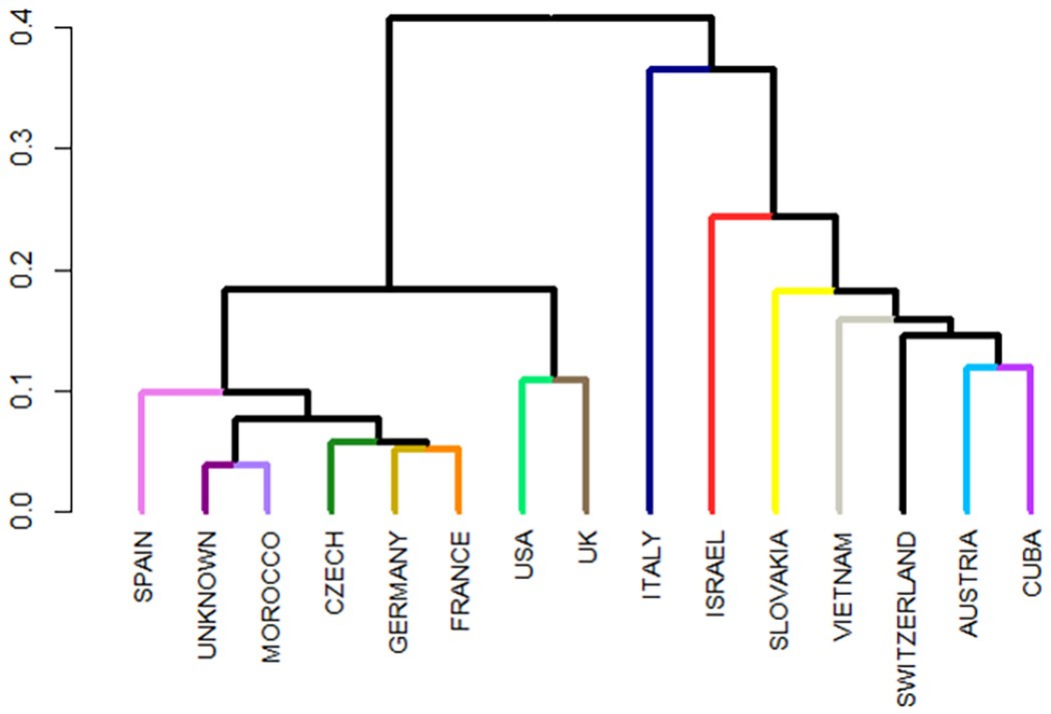
Diagram showing the structure of the pedigree file based on Wright’s fixation index (*F*_*ST*_).

In addition to the relatedness matrix calculated at the level of individuals, we also tested relations between subpopulations, defined by the location of individuals in zoological gardens. The dendrogram (Fig 3) was constructed based on Wright’s fixation index (*F*_*ST*_), which expresses the genetic similarity between subpopulations. *F*_*ST*_ decreases as inbreeding in the population increases—that is, the closely related animals are genetically similar because they come from a common ancestor. As shown in Fig 3, three main groups were identified. The shorter branches indicate greater relatedness, or a more inbred population. Among the countries, Italy showed the greatest genetic differentiation and was completely isolated from the others, but this outcome is because the studbook of Italian zoos lists only one male, which has not been involved in reproduction. On the contrary, Morocco and the unknown location showed negligible genetic differentiation (< 0.05), which can also be explained by the lack of information about these individuals. In Morocco, the zoo keeps a studbook, and all individuals that were included in this analysis are putative representatives of the ‘Moroccan Royal lions’. These countries, together with Spain, the Czech Republic, France and Germany, showed lower *F*_*ST*_ values, which clearly indicate that individuals in these zoos are more related and have been frequently involved in reproduction. The division of the population into individual clusters also reflects the global breeding plan of the ‘Moroccan Royal lions’. On the contrary, countries with higher *F*_*ST*_ values (> 0.1) are placed in the third group. These countries have individuals characterised by a lower inbreeding as well as a lower reproductive capacity.

The results according to the PCA and *F*_*ST*_ are consistent, as both analyses point to the creation of three separate groups. When compared with the results reported by Dures et al. [91], we can state that the reference population of the ‘Moroccan Rabat Zoo lineage’ faces a higher risk of inbreeding depression than its historical population. There are several examples in farm animals that are free of inbred depression, such as English thoroughbreds, Arabian horses and Japan Wagyu cattle, which have been bred long term based on the basis of mating of close relatives. The process is known as a so-called ‘genetic cleaning’. The population is homozygous, but at the same time, all lethal and sublethal factors are gone from the population. In the ‘Moroccan Royal lions’ population, inbreeding depression has begun to manifest itself in the form of musculoskeletal problems and poor or no breast milk production. In some cases, this has resulted in hormonal and reproductive problems (aspermatic males, infertile females) [53–56, 95, 96].

**Fig 4:**
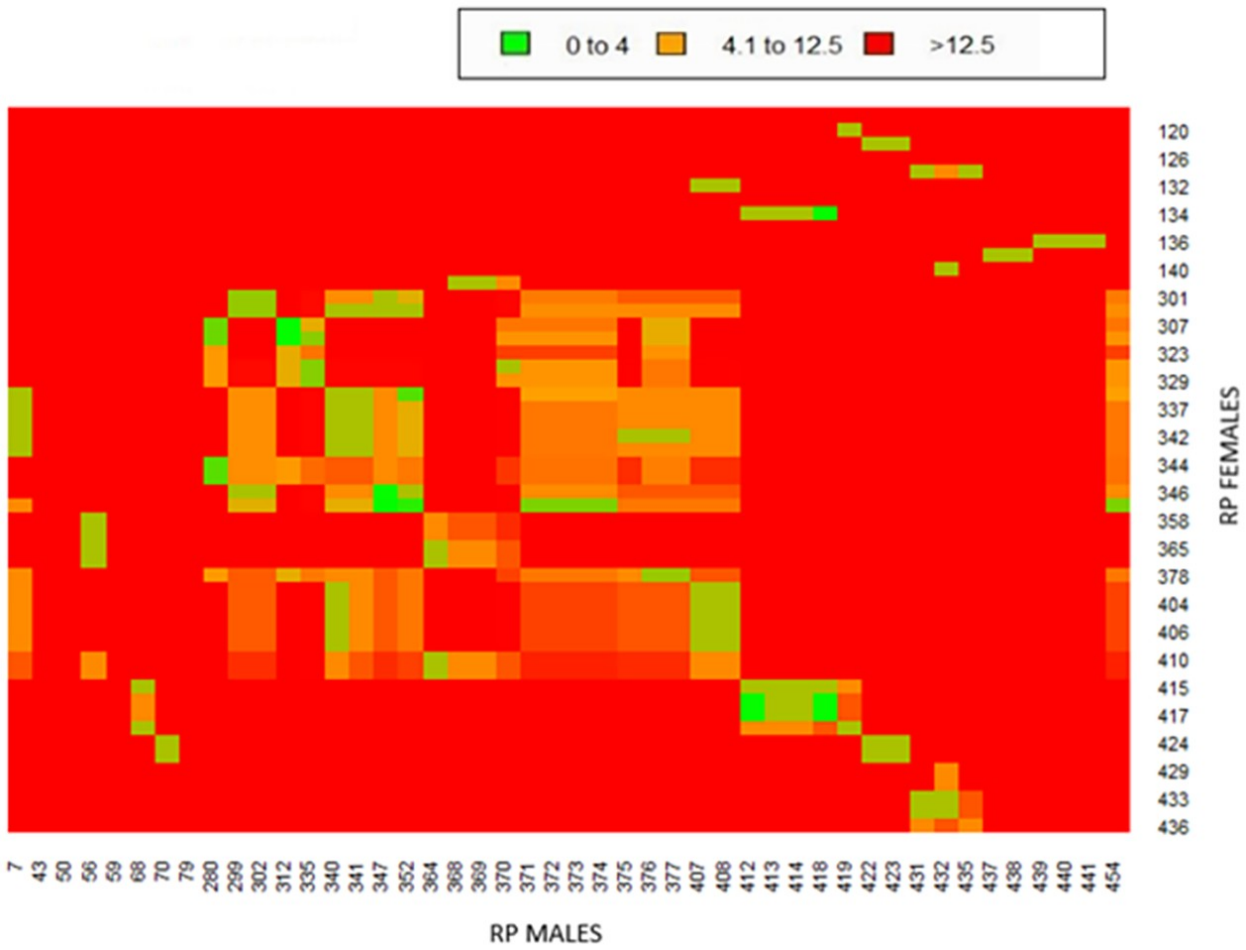
Diagram showing the average of relatedness among individuals from the reference population.

**Fig 5:**
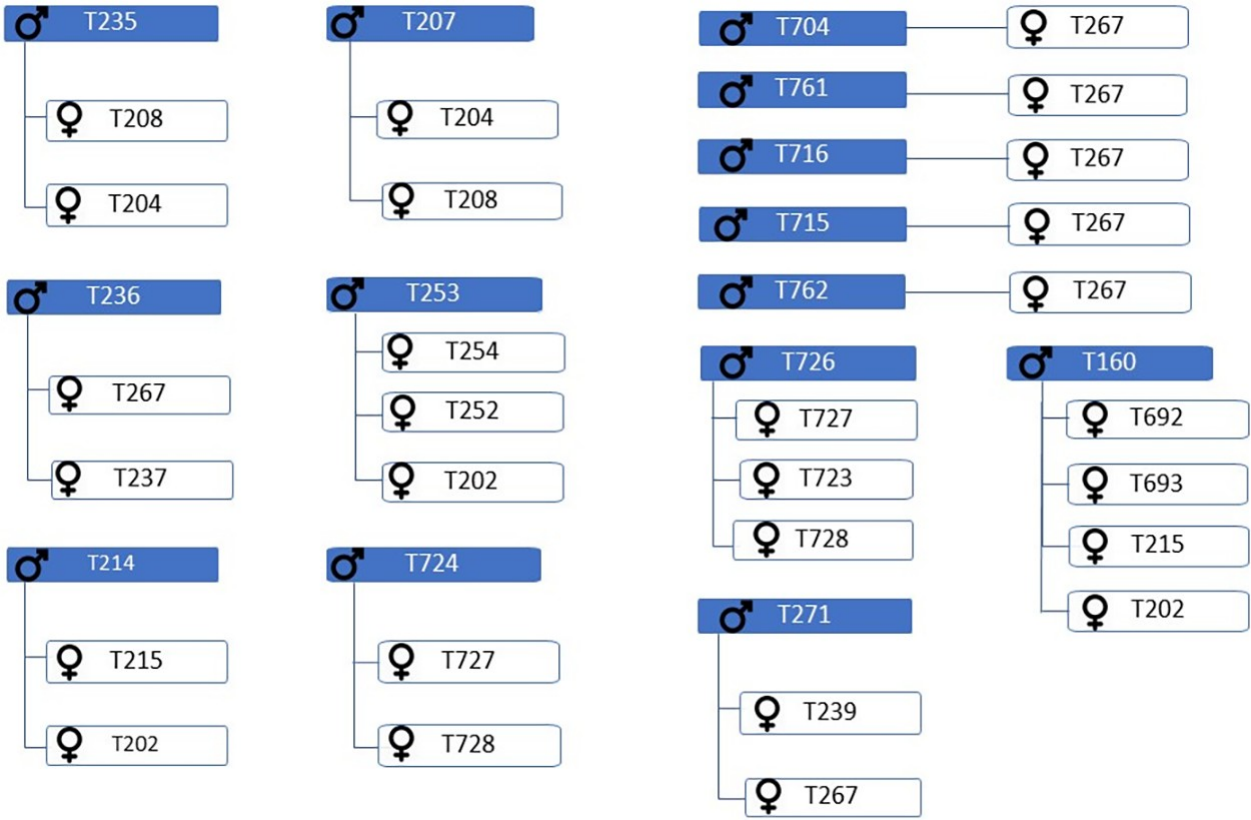
Representative mating plan of pairs suitable for reproduction. T—genus designation in the studbook; ♂—male; ♀—female.

One of the ways to avoid reproduction problems in population is the optimisation of mating plans with a specific focus on rate of inbreeding and average relatedness among animals. Fig 4 shows a heatmap regarding the average relatedness among animals in the reference population capable of reproducing. According to the average relatedness coefficient, the animals were divided into three groups (in Fig 4 coloured by green, orange and red depending on the *AR* value). The largest group comprises individuals whose *AR* value is > 12.5%. The second group consisted of individuals whose average relatedness is 4%-12.5%. The third group has individuals with *AR* < 4%; these are the only individuals that can be considered most suitable for reproduction schemes—that is, mating programmes should be based preferentially on those individuals. There are only 30 individuals in this group (Fig 5). In the future mating plan, it would also be advisable to use individuals from the second group, but only those with a lower average relatedness (AR close to 4%).

Black [5] stated that the genome of the Barbary lions as wild ancestors of several captive lion populations should be precisely determined before we turn to an alternative source of animals. Barnett et al. [90] pointed to one possibility of preserving lions *in situ* in North Africa (based on the assumption that the ‘Moroccan Royal lions’ have a close genetic affinity to Asian lions) using Asian lions to reintroduce lions of North African origin. The Asian lion population surviving today in India is restricted to Gir National Park and the surrounding areas in the Indian state of Gujarat. Because the origin of the Gir population and their relationship to the Barbary and ‘Moroccan Royal lions’ has not yet been fully confirmed, these animals have not been included in this study to prevent bias. If the Moroccan lions turn out to be related to the Indian lions, then the possibility of using the Gir population could be reconsidered.

## Conclusions

Increased values of pedigree completeness indices in the reference population point to a gradual improvement in the quality of pedigree information. Although it is well known that the pedigree data and studbook of the ‘Moroccan Rabat Zoo lineage’ is not accurate for all historical stages, it provides valuable information about the current population of the ‘Moroccan Royal lions’.

The results indicate the high value of inbreeding in the observed captive population of ‘Moroccan Royal lions’. Considering the risk of an inbreeding increase in future generations, the formation of mating plans for individuals with an *F* value > 1% will be essential. Ancestors that are currently considered founders (based on breeding records) do not have precisely defined relationships with other ancestors. For this reason, it is not possible to rule out that the founders had already contributed to a certain percentage of the inbreeding that was later passed on to progeny. The results point to the genetic fragmentation of three groups, which are not based on political, or geographical boundaries.

For this reason, a higher average coefficient of relatedness can be expected compared with the average values of the inbreeding coefficient observed in both the reference population and the pedigree file. The average inbreeding coefficient values in the reference population were low compared with the pedigree file. The results from the relationship matrix point to individuals ideally suited for reproduction (with *AR* < 4%). This small group of 30 animals suitable for the mating programme could also be enlarged with the individuals from the second group (with *AR* up to 12.5%). However, individuals should be selected primarily when they have a lower relatedness value. The breeding programme should include an assessment of relatedness to maximise genetic diversity.

Conservation programmes and appropriate mating plans are needed to control the inbreeding in the ‘Moroccan Royal lions’ among zoos worldwide. However, given the small number of individuals currently living in captive collections worldwide, an increase in the intensity of inbreeding due to the mating between more closely related animals can be expected in the future. Therefore, mating programmes oriented towards the concentration of base ancestor genomes in the progeny generations could be a suitable strategy for the future preservation of ‘Moroccan Royal lions’. Perhaps most importantly, the population of ‘Moroccan Royal lions’ could contribute to preserve in captivity and potentially *in situ* the highly endangered northern lion subspecies *P*. *leo leo*, which is currently suffering across the last fragmented areas of its previous historic territory of Western Africa, North Africa, the Middle East and India. In our view, now is the time to include the ‘Moroccan Royal lions’ group within the strategic conservation of lions and to engage the zoo community in active, systematic management of the population to preserve genetic diversity and viability for the future.
